# Promising Role of Fruitless Wolfberry Bud Tea in Combating *Nakaseomyces glabratus* Resistance

**DOI:** 10.3390/pathogens14040351

**Published:** 2025-04-04

**Authors:** Liping Zhang, Zhiyan Ma, Xuezhang Zhou, Ziping Zhang, Tao Wu

**Affiliations:** 1 Department of Clinical Laboratory Medicine, People’s Hospital of Ningxia Hui Autonomous Region, Ningxia Medical University, Ningxia Hui Autonomous Region, Yinchuan 750002, China; zhanglipingxly@163.com; 2 College of Life Science, Key Laboratory of Ministry of Education for Protection and Utilization of Special Biological Resources in Western China, Ningxia University, Yinchuan 750021, China; amzy123456782020@outlook.com (Z.M.); zhouxuezhang@nxu.edu.cn (X.Z.)

**Keywords:** fruitless wolfberry bud tea, *N. glabratus*, antifungal effect, drug efflux pumps

## Abstract

The rising antifungal resistance in *Nakaseomyces glabratus*, especially to azole drugs like fluconazole, itraconazole, and voriconazole, presents a significant clinical challenge. Plant-derived compounds with synergistic antifungal effects offer a promising solution. Fruitless wolfberry bud tea, rich in flavonoids from a *Lycium barbarum* L. hybrid, shows potential but is underexplored in antifungal therapies. This study assessed FWE’s antifungal efficacy alone and with azoles against resistant *N. glabratus* isolates, exploring mechanisms like efflux pump inhibition and gene expression changes. A total of 52 clinical isolates were tested. Fruitless wolfberry bud tea was methanol-extracted (FWE) and lyophilized. Antifungal susceptibility was evaluated using broth microdilution, and synergistic effects were analyzed with checkerboard assays. Growth inhibition, rhodamine 6G efflux, and qRT-PCR for resistance-related genes were conducted. FWE demonstrated inhibitory activity with MICs ranging from 16 to 32 μg/mL. When combined with ITR or VRC, synergistic or additive effects were observed, reducing MICs by 2–8-fold. FWE + VRC exhibited synergy (FICI ≤ 0.5) in 50% of isolates, while FWE + ITR showed synergy in 37.5%. Efflux pump activity, measured by rhodamine 6G, significantly decreased in combination groups (11.4–14.6%) compared to monotherapy (17.3–17.5%). qRT-PCR indicated downregulation of *CgCDR1*, *CgERG11*, and *CgPDR1* in FWE-treated Cg 1 isolate, with greater suppression in combination groups. FWE might boost the bacteriostatic impact of azole antifungal drugs by blocking efflux pumps and altering the expression of resistance genes. This study identifies FWE as a potent adjuvant to overcome cross-resistance, supporting its inclusion in antifungal strategies. Further research to identify bioactive compounds in FWE and in vivo validation is necessary for clinical application.

## 1. Introduction

*Nakaseomyces glabratus* (*Candida glabrata*) is a haploid yeast species within the *Nakaseomyces* clade, and it is differentiated from other *Candida* species, such as *Candida albicans*, by its diploid genome and its inability to form true hyphae [1]. *N. glabratus* infection rates have recently increased, particularly among immunocompromised individuals [2,3]. The latest data from the World Health Organization indicate that invasive candidiasis caused by *N. glabratus* is a highly severe condition, with an all-cause mortality rate of 20–50% at 30 days [4]. The risk of infection is predominantly associated with factors that weaken host immunity. *N. glabratus* ’s pathogenicity arises from several virulence factors, such as adhesins for epithelial adherence, biofilm formation, and hydrolytic enzyme secretion. Unlike *C. albicans*, *N. glabratus* employs unique immune evasion tactics: reducing β-glucan exposure to avoid host recognition, altering mannoprotein structures to suppress NLRP3 inflammasome activation, and lacking hyphal morphogenesis to prevent pyroptosis [5,6]. Meanwhile, it is important to highlight that recent research indicates co-infection with gastrointestinal microbiota may enhance the dissemination of *N. glabratus* [7].

Furthermore, resistance to antifungal treatments in *N. glabratus* has grown, notably against agents like fluconazole (FLC), itraconazole (ITR), and voriconazole (VRC) [8,9]. This resistance, typically arising from prolonged exposure to such drugs, reduces treatment effectiveness and heightens mortality rates [10]. In clinical settings, *N. glabratus* infections are frequently associated with co-infections with other *Candida* species, hence complicating the treatment regimen [11]. Therefore, early detection of *N. glabratus* and implementation of suitable antifungal treatments are of the utmost importance [4,8].

Numerous studies have demonstrated that certain plant-derived compounds exhibit significant anticandidal activity and can boost the effectiveness of antifungal agents against *Candida* species when used synergistically with antifungal drugs, particularly azoles [12,13,14]. *Lycium barbarum* L., commonly called goji or wolfberry, is a prominent species in traditional Chinese medicinal flora. Mature fruits have been a component of traditional Chinese medicine for treating various illnesses for more than two thousand years [15,16]. In regions including China and Southeast Asia, goji leaves are extensively utilized in various forms—such as tea, culinary vegetables, and herbal preparations—and are esteemed for their wide array of pharmacological properties, which encompass antimicrobial, antioxidant, and antidiabetic effects [17,18,19,20]. Research has demonstrated that goji leaf extract contains higher concentrations of flavonoids and phenolic acids, including chlorogenic acid, quercetin, and rutin, than its fruit and root bark extracts [21,22]. Furthermore, both in vivo and in vitro studies have demonstrated that specific flavonoids, including quercetin, kaempferol, and rutin, exhibit multiple antifungal properties against *N. glabratus*. These effects include: (1) inhibiting CYP51 to disrupt ergosterol biosynthesis; (2) suppressing Cdr1/Cdr2 drug efflux pumps; and (3) reducing biofilm formation by downregulating adhesins [23,24]. Consequently, flavonoids are promising natural therapeutic agents for treating candidiasis-related diseases.

**Figure 1:**
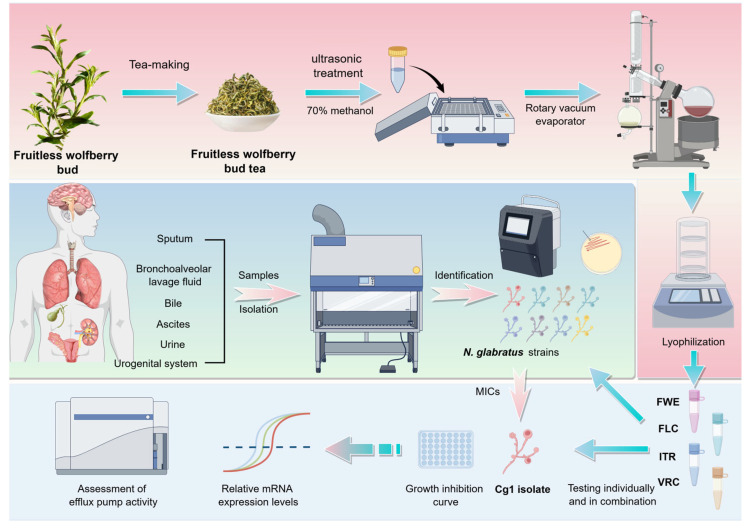
The methodology of this study encompasses extracting fruitless wolfberry bud tea, identifying and purifying clinical isolates, individual and combinatorial drug testing, and assessing efflux pump activity.

The fruitless wolfberry, a high-quality leafy goji berry variety, results from interspecific hybridization between local wild wolfberry species and *Lycium barbarum* L. cultivar. This variety does not flower or bear fruit, leading to a concentration of nutrients in tender buds, thereby providing exceptional nutritional value [25]. The fruitless wolfberry exhibited significantly higher total flavonoid content and more robust free radical scavenging activity than the other cultivars. The production of fruitless wolfberry bud tea involves harvesting tender buds from the plants, followed by a series of intricate processing steps aimed at preserving bioactive compounds, particularly total flavonoids, within the leaves [25]. Given the bioavailability and minimal toxicity of this plant source, along with the substantial total flavonoid content of fruitless wolfberry bud tea and the emerging threat posed by *N. glabratus*, this investigation sought to determine the efficacy of fruitless wolfberry bud tea extract (FWE) as a monotherapy and in combination with azole antifungal agents against azole-resistant clinical strains of *N. glabratus*. Additionally, we aimed to elucidate the mechanisms underlying any observed interaction effects between FWE and VRC or ITR using a rhodamine 6G efflux assay and quantitative reverse transcription polymerase chain reaction (qRT-PCR) (Figure 1).

## 2. Materials and Methods

### 2.1. Strains

A total of 52 clinical isolates of *N. glabratus* were identified and collected by the Clinical Laboratory of the People’s Hospital of Ningxia Hui Autonomous Region, comprising 18 isolates from sputum, 11 from bronchoalveolar lavage fluid, 12 from urogenital specimens, 6 from urine, 3 from bile, and 2 from ascitic fluid. Initially, colony characteristics were identified on Chromogenic Candida agar using standard clinical microbiological techniques (Chromagar, Paris, France). Subsequently, the isolates were verified using the VITEK2-compact automated bacterial identification system. *C. parapsilosis* ATCC 22019, used as a reference strain, was supplied by the Center for Clinical Laboratories of China’s National Health Commission. For purity and viability assurance, isolate stocks were preserved at −80 °C and subcultured at least twice before conducting experiments. Before conducting each experiment, the test strains were cultured in Modified Sabouraud dextrose Agar Medium (Hope Bio-Technology Co., Ltd., Qingdao, China, composition: 2% maltose, 1% peptone, 1.8% agar) at 37 °C for 48 h under aerobic conditions. Subsequently, fungal cells in the exponential growth phase were harvested and mixed in RPMI 1640 medium to obtain the desired concentrations for further experiments.

### 2.2. Chemicals

The Fruitless Wolfberry Bud Tea was sourced from Ningxia Qiya Food Technology Co., Ltd. (Zhongwei, China. Product Standard Code: Q/QYKJ00025, Production Date: 20230805). The fluconazole (FLC) and itraconazole (ITR) were procured from MeilunBio^®^ Co. (Dalian, China), and voriconazole (VRC) was procured from Shanghai Yuanye Bio-Technology Co. in Shanghai, China. These azole antifungal agents were prepared as a stock solution in dimethyl sulfoxide (DMSO) at a concentration of 16 mg/mL. The solutions were kept at −20 °C and diluted in RPMI 1640 medium prior to use.

### 2.3. Preparation of the FWE

Pulverized tea samples (10.0 g) were combined with 150 milliliters of 70% methanol and subjected to ultrasonic treatment. Ultrasound-assisted extraction was performed using a Kedao 350 Ultrasonic Instrument (Shanghai, China), operating at a frequency of 35 kHz and an intensity of 150 W/cm², with 5-s pulse intervals over a 30-min period, while the samples were maintained in an ice bath to prevent thermal degradation. The resulting homogenate was subsequently filtered through a 0.45-µm polyvinylidene fluoride (PVDF) membrane (Millipore^®^, Billerica, MA, USA) to remove cellular debris. This was followed by rotary evaporation using a Rotavapor R-215 system (Büchi Labortechnik AG, Flawil, Switzerland), set at 40 °C, 35 mbar, and 80 rpm, to concentrate the extract to a 10% (*w*/*v*) dry mass. The concentrated extract was lyophilized and stored at −20 °C for subsequent use.

The fruitless wolfberry bud tea extract (FWE) was prepared as a 16 mg/mL stock solution in DMSO, stored at −20 °C, and freshly diluted to the needed concentrations using a culture medium before application. To ensure no cytotoxic effects, the DMSO level was maintained at under 0.1%.

### 2.4. Evaluating Susceptibility to Antimicrobial Agents

The broth microdilution method, as outlined in the Clinical and Laboratory Standards Institute (CLSI) guidelines M27-A3, was used for antimicrobial susceptibility testing [26], using sterile 96-well microtitration plates.

During the exponential growth phase, 100 μL of *Candida* cells (1.5 × 10^3^ cells/mL) in RPMI 1640 culture medium were combined with 100 μL of RPMI 1640 broth containing the test agents at two-fold serial dilutions. The final concentrations for each drug were as follows: FLC at 128 to 0.25 μg/mL, VRC at 16 to 0.0156 μg/mL, ITR at 32 to 0.0313 μg/mL, and FWE at 512 to 1 μg/mL. Control wells contained 200 μL of fungal suspension in RPMI 1640 without the test agent as the growth control and 200 μL of RPMI 1640 medium as the blank control. The inhibition of growth was evaluated after a 24-h incubation period at 35 °C. Antifungal activity was assessed using the minimum inhibitory concentration (MIC), defined as the lowest concentration of the drug that prevents visible growth, as observed visually. All assays were conducted in triplicates. The classification of strains into susceptible-dose dependent (SDD) or resistant was based on clinical breakpoints (CBPs) for FLC, with MICs ≤ 32 μg/mL for SDD and MICs ≥ 64 μg/mL for resistance. There are no established CBPs for VRC and ITR; therefore, strains were classified as non-wild-type (NWT) if the MIC of VRC was greater than 0.25 μg/mL or the MIC of ITR was more than 4 μg/mL, using species–specific epidemiological cut-off values [27], while lower MIC values indicated wild-type (WT).

The synergistic interaction between FWE and azoles was assessed using the checkerboard titration method [28]. This method involves adding combinations of the two test agents, each subjected to a two-fold serial dilution, to the wells of a sterile 96-well plate. Each well was filled with 100 μL, containing 1.5 × 10^3^ cells/mL. In RPMI 1640 medium, the test agents underwent a two-fold serial dilution to achieve final concentrations from four times the MIC to 1/32 of the MIC. Wells in rows A–H received a 50 μL sample from the FWE dilution series, while wells in columns 1–8 were filled with a 50 μL sample from the azole drug dilution series. Subsequently, each well received 100 μL of cell suspension, resulting in a final concentration of 1.5 × 10^3^ cells/mL, except for columns 9–12. Column 10 received 200 μL of cell suspension to act as a growth control, and column 12 was given 200 μL of RPMI 1640 medium to serve as a negative control. The plates were then incubated at 35 °C for 24 to 48 h.

In vitro assessment of the drug combination interaction was conducted using the fractional inhibitory concentration index (FICI) [29], which was calculated as follows: FICI = FIC_A_ + FIC_B_ = C_A_/MIC_A_ + C_B_/MIC_B_. In this scenario, MIC_A_ and MIC_B_ denote the MICs of drugs A and B when used on their own, while C_A_ and C_B_ signify the MICs when the drugs are combined. The FICI values were understood as follows: synergy is indicated by a FICI of 0.5 or less; additivity is shown by a FICI greater than 0.5 but less than 1.0; indifference is indicated by a FICI from 1 to less than 4.0; and antagonism is shown by a FICI of 4.0 or more.

### 2.5. Antifungal Curve of Tested Agents Against N. glabratus

To determine the impact of FWE and azole antifungal agents together on inhibiting the growth of *N. glabratus*, antifungal assays were conducted on the Cg1 isolate, NWT resistant to ITR and VRC, as the study model. Growth inhibition experiments were organized into six categories: (I) control without drugs, (II) FWE only, (III) ITR by itself, (IV) VRC alone, (V) FWE combined with ITR, and (VI) FWE combined with VRC. All test agents were administered at a concentration of 1/2 × MIC.

In summary, cells of *Candida* in the period of exponential expansion were collected and standardized to a density of 1.0 × 10^7^ cells per mL using RPMI 1640 medium. Subsequently, a volume of 1 mL of the fungal suspension was introduced into flasks with 100 mL of RPMI 1640 medium. The test agent was incorporated into the culture to attain a final concentration equivalent to half of the MIC. Following this, the cultures were relocated to a shaker set to 35 °C and agitated at 120 rpm for 48 h. Throughout the cultivation period, samples were taken from each group at specific time points (0, 3, 6, 9, 12, 24, 36, and 48 h after incubation) to assess optical density (OD) at 600 nm. Growth curves for the Cg1 isolate in the presence and absence of test agents were constructed to illustrate the relationship between culture time (h) and OD values [30].

### 2.6. The Examination of Rhodamine 6G Efflux

Efflux pump activity was assessed through flow cytometry using rhodamine 6G [31]. The procedure was adapted from the method described in [32] with minor modifications. In summary, the overnight-cultured isolates were diluted to 5 × 10^7^ cells/mL using 2 mL of phosphate-buffered saline and maintained at 30 °C and shaken at 200 rpm for 4 h to induce deprivation. Subsequently, rhodamine 6G was introduced to reach a concentration of 10 μM, and the mixture was stirred at 30 °C for two hours. Later, the culture was placed on ice to inhibit rhodamine 6G uptake. Then, the cells underwent two washes with cold, sterile PBS after being collected. An aliquot of 50 μL cells were re-dispersed in PBS buffer and subjected to centrifugation at 3000 rpm for 15 min. After discarding the supernatant, the cells were reintroduced into PBS. A BD FACS Aria^TM^ II flow cytometer (BD Biosciences, San Jose, CA, USA) was used to measure the cells’ fluorescence uptake at 535 nm. Fluorescence intensity represents the initial fluorescence before efflux. Simultaneously, two 50 μL aliquots of the cell suspension were prepared. One aliquot was combined with 1 mL of glucose-free PBS, while the other was mixed with 1 mL of PBS containing glucose with a terminal concentration of 4 mmol/L. Each preparation was incubated at 30 °C and shaken at 200 rpm for 2 h. Following this, the cells’ fluorescence was evaluated, and the resulting fluorescence intensity indicated the fluorescence level after efflux in the presence or absence of glucose. Cells that were not exposed to rhodamine 6G acted as the control group.

### 2.7. Gene Expression Measurement Using qRT-PCR

The gene expression levels potentially associated with drug resistance, specifically efflux pumps (*CgCDR1*, *CgCDR2*, *CgSNQ2*, and *CgPDR1*) and azole targets (*CgERG11*), were quantified using qRT-PCR. This analysis examined variations in gene expression under different treatment conditions, including FWE, ITR, or VRC alone, and the combination of FWE and ITR or VRC. The Cg1 isolate was cultured in RPMI 1640 medium until it achieved the middle stage of exponential growth. Next, the cells were shifted to a new medium (control) or a new medium supplemented with the tested drugs at half the MIC (1/2 × MIC) until the OD at 600 nm reached a standard range of 1.5 to 2.5. Then, the cells were harvested by centrifugation. For RNA extraction at a later stage, the samples were frozen right away at −80 °C. TRIZOL reagent purchased from TIANGEN Biotechnology Co. (Beijing, China) was used to extract total RNA from the harvested cells. The synthesis of complementary DNA was performed using HiScript^®^ II Q RT SuperMix R223 from Vazyme Biotechnology Co. (Nanjing, China). The qPCR was conducted using ArtiCanATM SYBR qPCR Mix (TSE501, TSINGKE, Beijing, China) on an ABI 7500 Real-Time PCR System (Applied Biosystems, New York, NY, USA). All procedures strictly adhered to the manufacturer’s protocol. Quantification of target gene expression levels was performed using the 2^−ΔΔCT^ method, with 18S rRNA as the internal reference gene. Each experiment was carried out in three separate trials. Primers for the target genes are detailed in Table 1, the design and synthesis of all primers were carried out by Sangon Biotech in Shanghai, China.

### 2.8. Statistical Analysis

GraphPad Prism software 10.1.2 facilitated the statistical analysis and visualization of the data. An ANOVA, incorporating Tukey’s correction for multiple comparisons, was utilized to assess differences among multiple groups, with statistical significance established at a P-value of less than 0.05. Figure 1 was created utilizing Figdraw (https://www.figdraw.com/static/index.html#/ (accessed on 8 November 2024).

## 3. Results

### 3.1. Antifungal Susceptibility Testing

According to the CLSI interpretive criteria, all 52 isolates were classified as FLC SDD with MICs of ≤ 32 μg/mL. Among these, 5.7% (three of fifty-two) and 15.4% (eight of fifty-two) of the isolates were identified as NWT for ITR (MICs > 4 μg/mL) and VRC (MICs > 0.25 μg/mL). The three isolates NWT for ITR were also NWT for VRC. These findings suggest that the *N. glabratus* clinical isolates tested were cross-resistant to azole antifungals. Following a thorough analysis of the MICs for fifty-two clinical isolates of *N. glabratus*, eight isolates (designated as Cg1-Cg8) exhibiting elevated MIC values for three azole drugs were selected for further experimentation.

### 3.2. Investigation of the Interaction of FWE with Azole Antifungals

We examined the interaction between FWE and three azole antifungal agents against eight NWT isolates for VRC. The results demonstrated that FWE alone exhibited growth-inhibitory activity against eight clinical isolates of *N. glabratus*; MIC values fall within the range of 16 to 32 μg/mL. Furthermore, when combined with azole agents, a 2- to 4-fold decrease in the MIC is noted, depending on the specific antifungal agent and the strain being analyzed.

According to the FICI value, the interaction was classified [29]. Table 2, Table 3 and Table 4 demonstrate the synergistic, additive, and indifferent interactions when combining FWE with antifungal agents, with no antagonistic effects observed across all strains. Table 2 illustrates the additive interactions for seven isolates and an indifferent interaction for one isolate (FICI = 1.0). The MIC values for FLC alone ranged from 8 to 32 μg/mL for all eight isolates. When combined, the reduction in MICs for FLC ranged from two- to eight-fold depending on the strain; while for FWE, it was four- to eight-fold. (MIC = 4–16 μg/mL).

Table 3 demonstrates a synergistic interaction with three isolates (Cg1, Cg3, and Cg7), with a FICI of 0.375, while the remaining five isolates displayed an additive effect, with FICI values between 0.625 and 0.75. Across all eight strains, ITR alone showed MIC values from 0.5 to 8 μg/mL, with three isolates identified as NWT for ITR. The MICs for these isolates significantly decreased from 2- to 8-fold when ITR was used in combination. These findings suggest that the combination of FWE with ITR exhibits a synergistic or additive effect across all tested *N. glabratus* isolates, potentially reducing the required dosage of ITR.

Table 4 illustrates a synergistic effect for four isolates with an FICI ranging from 0.375 to 0.5; while, the other four isolates displayed an additive effect, each with a FICI of 0.75. Specifically, for isolates Cg1 and Cg6, the combination of FWE and VRC demonstrated a synergistic interaction (FICI = 0.375); the MIC of VRC was reduced by eight times and the MIC of FWE by four times compared to when they are used separately. Similarly, for isolates Cg2 and Cg8, a synergistic effect of FWE with VRC was also evident (FICI = 0.5), leading to a four-fold decrease in the MIC of VRC in combination compared to its MIC when used alone.

### 3.3. Determination of Growth Inhibition Curve

Following an extensive analysis of the antimicrobial susceptibility results (MICs) and the interactions between FWE and azole antifungal agents, the clinical isolate Cg1 was chosen as the model strain for our subsequent investigations. Cg1, identified as *N. glabratus*, was isolated from the ascitic fluid of a patient in the intensive care unit who had been hospitalized for 18 days due to severe peritonitis and perforated peritoneum. Subsequent to the initial ascitic fluid culture, *N. glabratus* was also isolated from various other sites, including sputum, urine, and secretions, from the same patient.

**Figure 2:**
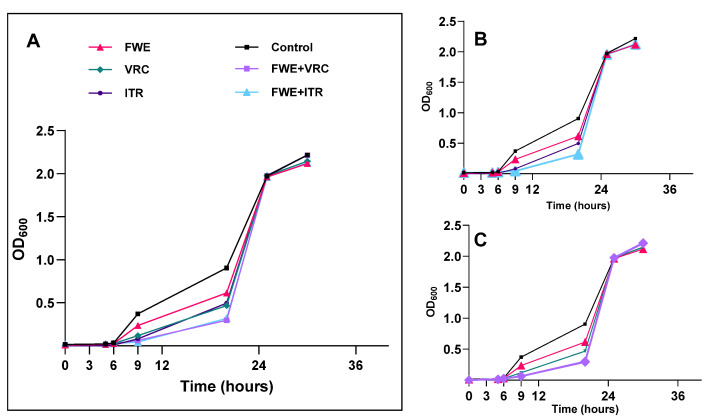
Growth inhibition curves of FWE, ITR, and VRC (alone and combined) against Cg1 isolate. (**A**) Temporal progression of fungal growth was examined with and without FWE, ITR, and VRC (alone and combined). (**B**) Inhibition curves of fungal growth for FWE and ITR (alone and combined). (**C**) Inhibition curves of fungal growth in FWE and VRC (alone and combined).

In the presence and absence of the tested agents, the temporal progression of fungal growth was examined to confirm the antifungal efficacy of FWE in combination with ITR or VRC against *N. glabratus* isolates. The control group exhibited an S-shaped growth curve, with *N. glabratus* entering the logarithmic phase at 6 h and reaching the stationary phase at 24 h (Figure 2). Compared to the untreated control group, the growth curves of all drug-treated groups exhibited a rightward shift during the 6 to 24-h period. The growth curve of the combination drug group (FWE + ITR or FWE + VRC) demonstrated a significant rightward shift compared to those treated with FWE, ITR, or VRC alone. These observed alterations in the growth curves suggest that FWE alone, as well as ITR or VRC alone, possess antifungal activity capable of delaying the growth of clinical isolates of *N. glabratus*. The combination of FWE with ITR or VRC resulted in a significant enhancement of fungistatic activity compared to the effects of ITR or VRC alone, thereby confirming the synergistic growth-inhibitory effect of FWE when combined with ITR or VRC. Furthermore, the growth curve analysis indicated that FWE, ITR, VRC, and their combinations exclusively demonstrated fungistatic properties without exhibiting fungicidal effects.

### 3.4. Assessment of Efflux Pump Activity

Flow cytometry was used to assess the uptake and efflux of rhodamine 6G in *N. glabratus* isolate Cg1 subjected to various treatments (Table 5). In the absence of glucose, a negligible efflux of rhodamine 6G was detected across all treatment groups. Conversely, glucose administration resulted in elevated efflux levels in all the groups. Specifically, in the presence of glucose, the efflux levels for treatments with ITR or VRC alone (17.3% and 17.5%, respectively) were significantly higher than those observed in the other groups compared to the control group (9.1%). In the presence of FWE + ITR or FWE + VRC, efflux levels (11.4% and 14.6%, respectively) were higher than those in the control group. However, these levels were lower than when FWE or ITR/VRC were administered alone (Supplementary Figures S1–S3).

### 3.5. Determining Potential Drug-Resistance Gene Expression Levels

The relative mRNA expression levels of *CgCDR1*, *CgCDR2*, *CgERG11*, *CgPDR1*, and *CgSNQ2* in the clinical *N. glabratus* isolate Cg1 were assessed under various treatment conditions, including FWE, ITR or VRC alone, and the combination of FWE with ITR or VRC, using qRT-PCR.

**Figure 3:**
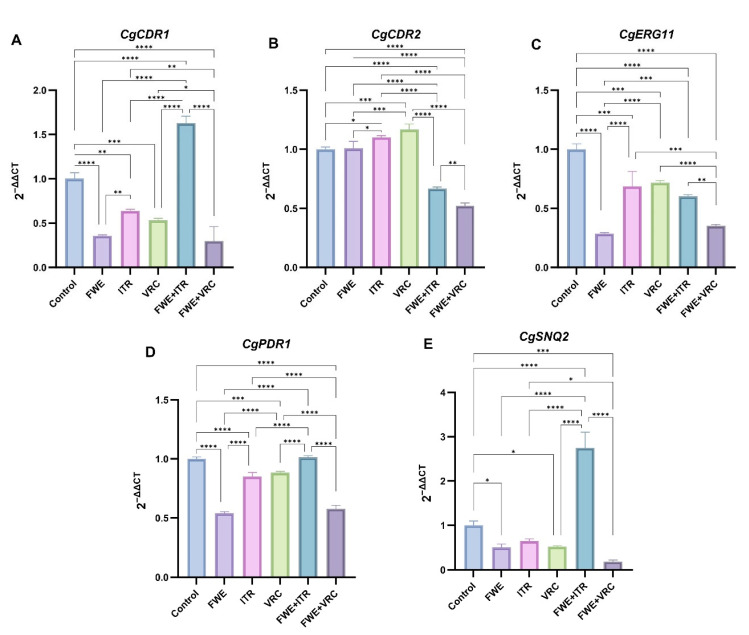
Relative mRNA expression levels of potential drug resistance genes. Relative mRNA expression levels of (**A**) *CgCDR1*, (**B**) *CgCDR2*, (**C**) *CgERG11*, (**D**) *CgPDR1*, and (**E**) *CgSNQ2* were quantified using qRT-PCR in the clinical *N. glabratus* isolate Cg1 under treatment with FWE, ITR, or VRC (alone and combined. The data represent the mean values derived from three independent experiments. The control isolate was not exposed to any drugs. Statistical significance: * *p* < 0.05, ** *p* < 0.01, *** *p* < 0.001, **** *p* < 0.0001.

The qRT-PCR analysis revealed that the single-agent treatment groups FWE, ITR, and VRC significantly inhibited *CgCDR1* gene expression compared to the control group, with statistical significance levels of *p* < 0.0001, *p* < 0.01, and *p* < 0.001, respectively (Figure 3A). The inhibitory effect of FWE on *CgCDR1* gene expression was significantly greater than that of ITR (*p* < 0.01). Regarding drug combinations, the FWE + VRC group exhibited significantly inhibited *CgCDR1* gene expression compared to the control group (*p* < 0.0001). This effect was significantly different from that of VRC alone (*p* < 0.05), even though it wasn’t significantly distinct from FWE alone. The FWE + ITR group exhibited significantly upregulated *CgCDR1* gene expression compared to all other treatment groups (*p* < 0.0001).

Figure 3B illustrates that the relative expression levels of the *CgCDR2* gene in the single-agent FWE-treated group did not show significant differences compared to the control group. Nevertheless, the expression levels were greater in the VRC- and ITR-treated groups than in the control group (*p* < 0.05, *p* < 0.001). The combined treatment groups (FWE + VRC and FWE + ITR) significantly inhibited *CgCDR2* gene expression compared to the control and single-agent groups (*p* < 0.0001). Furthermore, *CgCDR2* gene expression differed significantly between the FWE + VRC and FWE + ITR treatment groups (*p* < 0.01).

In Figure 3C, there is a marked reduction in *CgERG11* expression across all treatment groups relative to the control group, with the most substantial downregulation occurring in the FWE-treated group (*p* < 0.0001). Regarding drug combinations, FWE + ITR and FWE + VRC significantly inhibited *CgERG11* gene expression compared to the control group (*p* < 0.0001). The FWE + VRC treatment group demonstrated a significantly greater inhibitory effect than the FWE + ITR treatment group (*p* < 0.001), as well as the VRC (*p* < 0.0001) and ITR (*p* < 0.001) single-agent groups. However, the inhibitory effect was slightly lower in the FWE + VRC treatment group than in the FWE single-agent group, although this difference did not achieve statistical significance.

The single-drug treatment groups (FWE, ITR, and VRC) demonstrated significant inhibitory effects on *CgPDR1* gene expression compared to the control group (*p* < 0.0001, *p* < 0.0001, and *p* < 0.001, respectively; Figure 3D). The inhibitory effect of FWE on *CgPDR1* gene expression was significantly different from that observed with ITR and VRC (*p* < 0.0001 for both comparisons). Within the combination treatment groups, the FWE + VRC group significantly inhibited *CgPDR1* gene expression compared to the control group (*p* < 0.0001); however, this effect was slightly less pronounced than that observed with FWE monotherapy. Conversely, the FWE + ITR group displayed weak upregulation of *CgPDR1* gene expression compared to the control group, although this change was not statistically significant.

The single-drug treatment groups (FWE, ITR, and VRC) demonstrated varying levels of inhibition on *CgSNQ2* gene expression compared to the control group, with the FWE and VRC groups exhibiting more significant inhibition (*p* < 0.05 for both; Figure 3E). Regarding combination treatments, the FWE + VRC group significantly reduced *CgSNQ2* gene expression compared to the control group (*p* < 0.001). However, no significant difference was observed between the effects of FWE and VRC alone. Conversely, the FWE + ITR group significantly upregulated *CgSNQ2* gene expression, which was significantly higher than in all other treatment groups (*p* < 0.0001).

## 4. Discussion

Combination therapy is a promising strategy for enhancing the efficacy of antimycotic agents and mitigating the development of drug-resistant *Candida* species [33,34]. Several studies have indicated that various plant extracts exhibit intrinsic anti-*Candida* activity and synergistic effects when combined with established antifungal drugs [35,36,37]. However, most research has focused on combinations of FLC specifically targeting *C. albicans* [38,39]. Melchor-Martínez EM et al. [24] reported significant antifungal activity of three antifungal glycosylated flavonoids against clinical isolates of FLC-resistant *N. glabratus*. Flavonoids with higher antioxidant activities exhibited the greatest effectiveness against *N. glabratus*. Nonetheless, it remains unclear whether flavonoids can synergistically interact with azole drugs in clinical isolates of *N. glabratus*.

Goji leaves are extensively used as tea, vegetables, and herbs in China and Southeast Asia. Fruitless wolfberry is a superior leafy variety of goji berries, characterized by significantly higher total flavonoid content and enhanced free radical scavenging activity compared to other *Lycium barbarum* L. leaves [21]. According to Zhou et al. [40], fruitless wolfberry bud tea exhibits relatively higher antioxidant activity and greater total polyphenol content than *Lycium barbarum* L. leaf tea. This suggests that fruitless wolfberry bud tea is a valuable source of flavonoids and phenolic acids, including chlorogenic acid, quercetin, and rutin.

In this study, we examined the interactions of FWE with azole drugs against eight NWT clinical isolates of *N. glabratus* for VRC. The MIC values indicated that FWE alone exhibited antifungal activity against all eight isolates, with MIC values ranging from 16 to 32 μg/mL. When combined with azole drugs, particularly VRC or ITR, FWE demonstrated a strong inhibitory effect. Based on FICI, the combination of FWE and VRC exhibited a synergistic effect on four isolates and an additive effect on the remaining four isolates. This study demonstrated that combining FWE and ITR elicited a synergistic effect in two ITR NWT isolates and one ITR WT isolate. Conversely, no synergistic interaction was detected between FWE and FLC among the eight clinical isolates of *N. glabratus*, exhibiting an SDD response to FLC. The combination of FWE and FLC resulted in additive effects in seven isolates and an indifferent effect in one isolate. Both FWE + VRC and FWE + ITR combinations displayed synergistic interactions with the Cg1 isolate, which was NWT for VRC and ITR. Specifically, for the Cg1 isolate, MIC values for VRC and ITR were reduced by eight-fold, while the MIC for FWE was reduced four-fold when combined compared to its MIC alone. These findings indicated that FWE combined with VRC or ITR demonstrated a significant synergistic interaction with resistant isolates. Consequently, FWE has emerged as a promising synergistic agent to overcome cross-resistance to ITR and VRC in *N. glabratus*.

Rhodamine 6G, a fluorescent dye that shares a membrane transporter with azoles in certain *Candida* species [31], is frequently employed to evaluate efflux pump activity. In this study, clinical *N. glabratus* isolate Cg1 was used as a model to assess the efflux pump functionality. Rhodamine 6G efflux assay results indicated that cells cultured in the presence of ITR or VRC exhibited elevated efflux levels (17.3% and 17.5%, respectively) compared to the control group (9.1%), underscoring the significant role of efflux pump activity in the resistance of isolate Cg1 to ITR and VRC. In contrast, cells grown in the presence of FWE combined with ITR or VRC displayed reduced efflux levels compared to those exposed to ITR or VRC alone (11.4% and 14.6%, respectively). This finding suggests that combining FWE with ITR or VRC can inhibit the efflux of intracellular rhodamine 6G, with the observed synergistic effect associated with efflux inhibition in the isolate Cg1.

Numerous studies have underscored the considerable diversity in mechanisms conferring resistance to azoles [41,42,43]. Among these mechanisms, increased expression of efflux pumps, specifically those from the ATP-binding cassette (ABC) and major facilitator superfamily classes, has been linked to resistance against azoles [43]. In *N. glabratus*, most clinical resistance cases have been connected to the heightened activity of ABC transporters encoded by *CDR* genes [44,45]. Various reports have suggested that FLC resistance mechanisms in *N. glabratus* involve the overexpression of efflux pumps, such as *CgCDR1*, *CgCDR2*, and *CgSNQ2* [46,47,48]. *CgPdr1*, a zinc finger transcription factor, modulates the expression of these transporters, with *CgCDR1* and *CgCDR2* serving as the principal genes responsible for conferring azole resistance. According to Whaley et al. [49], CDR1 is the main transporter linked to PDR1 that causes multidrug resistance to azoles in the clinical isolate of *N. glabratus* being studied. Conversely, PDR1 and SNQ2 played a smaller role in azole resistance compared to CDR1. Yao et al. suggested that azole resistance in *N. glabratus* is primarily attributed to the sustained upregulation of the *CgCDR1* gene, while the *CgCDR2* gene plays a secondary role [47]. Furthermore, mutations that enhance the function of the *CgPDR1* gene, which encodes a zinc-finger drug resistance transcriptional activator, represent a major contributing factor to azole resistance in clinical isolates of *N. glabratus* [48]. Most of the existing research has focused on elucidating the mechanisms underlying FLC resistance, whereas comparatively fewer studies have investigated resistance to VRC or ITR in *N. glabratus*. Navarro-Rodríguez et al. [50] found no direct link between *ERG11* or efflux pump upregulation and VRC resistance but observed increased *ERG11* and *CDR1* expression in VRC-resistant strains.

This research examined how FWE, ITR, and VRC, both individually and together, influenced the expression of five drug efflux-related genes in a clinical isolate of *N. glabratus* Cg1 under different treatment scenarios. These findings indicate that treatment with FWE, ITR, or VRC alone upregulated the *CgCDR2* gene and downregulated *CgCDR1*, *CgERG11*, *CgPDR1*, and *CgSNQ2* genes relative to the control group. The FWE treatment alone significantly inhibited the expression of these four genes compared to ITR or VRC treatment alone. Furthermore, combining FWE and VRC significantly enhanced the downregulation of *CgCDR1*, *CgCDR2*, *CgERG11*, and *CgSNQ2* genes compared to VRC treatment alone. These results suggested that the synergistic interaction between FWE and VRC may be attributed to the downregulation of *CgCDR1*, *CgCDR2*, *CgERG11*, and *CgSNQ2* gene expression. However, this study did not establish a causal relationship between VRC resistance in clinically resistant strains and the expression of these genes. The concurrent administration of FWE and ITR led to a significant upregulation in the expression of the *CgCDR1*, *CgSNQ2*, and *CgPDR1* genes, exceeding the expression levels observed in both the control and individual drug treatment groups. Upregulation of *CgCDR1* and *CgSNQ2* was significantly pronounced. This observation indicates a substantial antagonistic effect on *CgCDR1* and *CgSNQ2* gene expression in clinically resistant strains when FWE and ITR are used concomitantly, increasing the mRNA transcripts of these two active exocytosis genes, thereby enhancing drug resistance. Overall, these findings align with existing literature on the relationship between drug efflux gene expression and drug resistance, underscoring the critical role of these genes in drug resistance development. Future research should aim to elucidate the direct connection between alterations in gene expression and drug resistance, as well as the underlying molecular mechanisms.

The clinical resistant strain Cg1 showed elevated efflux of intracellular rhodamine 6G and higher *CgCDR2* mRNA expression levels in ITR relative to the control group. It implies that *CgCDR2* has a greater impact on the Cg1 strain’s resistance to ITR than other genes. Similarly, our findings showed that intracellular rhodamine 6G efflux and *CgCDR2* mRNA expression were elevated with VRC compared to the control, suggesting a significant role of *CgCDR2* in Cg1’s resistance to VRC. Castanheira M et al. [46] observed a marked rise in the expression of *CgCDR1* and *CgCDR2* mRNA in 34 FLC-resistant strains. However, these findings do not provide evidence linking resistance to VRC or ITR with *CgSNQ2* gene expression. This is consistent with the data obtained by Yao et al. [47]. Increased expression of *CgCDR1* and *CgCDR2* genes was observed in the FLC-resistant group compared to the SDD group, whereas *CgSNQ2* expression remained unchanged. Furthermore, the *CgERG11* gene, another resistance-associated gene examined in this study, encodes the azole target and has been implicated in FLC resistance in *C. albicans* [51] but not in the azole resistance of *N. glabratus* [52]. These results do not provide evidence to determine whether resistance to ITR or VRC is linked to *CgERG11* gene expression. The development of resistance is influenced by a multitude of factors and cannot be attributed to a single resistance gene or mechanism. Consequently, further research is required to elucidate these complex interactions.

Conversely, this study revealed that the combination of FWE and VRC reduced the expression of five genes (*CgCDR1, CgCDR2*, *CgSNQ2*, *CgERG11*, and *CgPDR1*) compared to VRC treatment alone. This result suggested that the synergistic interaction between FWE and VRC may be attributed to the downregulation of these genes. In contrast, the combination of FWE and ITR decreased the expression of two genes (*CgCDR2* and *CgERG11*) compared to ITR alone, indicating that the synergistic effect between FWE and ITR may be associated with the downregulation of *CgCDR2* and *CgERG11*. These findings highlight the distinct mechanisms underlying the synergistic antifungal effects of FWE when combined with ITR or VRC. Even though the precise mechanism remains to be elucidated, our efflux pump inhibition assays and qRT-PCR data provide preliminary evidence suggesting that FWE may interfere with drug efflux systems. This mechanistic hypothesis necessitates further validation through proteomic and transcriptomic profiling in future studies. It is important to note, however, that a limitation of this study is the geographic heterogeneity of *N. glabratus* resistance, which was only superficially examined in representative resistant strains from the Ningxia Hui Autonomous Region. To thoroughly analyze the mechanism of FWE on clinically resistant strains of *N. glabratus*, it is essential to conduct comprehensive investigations in regions characterized by diverse host genetic backgrounds and medical practice patterns. Addressing these factors of geographic heterogeneity is crucial for future validation studies. This research suggests that combining FWE and azole antifungal agents reduces the required dosage and enhances the inhibitory efficacy of VRC or ITR against clinically resistant *N. glabratus*. However, the specific compounds responsible for this antifungal activity have yet to be identified, warranting further investigation.

## Figures and Tables

**Table 1:** Primers employed in qRT-PCR.

Genes	Nucleotide Sequence (5′-3′)
*18SrRNA-*F	TCTTTCTTGATTTTGTGGGTGG
*18SrRNA*-R	TCGATAGTCCCTCTAAGAAGT
*CgCDR1-*F	TAACCAGGTGGCAGAAGCAG
*CgCDR1-*R	CCACAAGTCAGGTTTGCAGC
*CgCDR2-*F	CAACGCTATGAGGGAAAA
*CgCDR2-*R	AACATAAGTGGCGTGGGT
*CgPDR1-*F	AACAGCTTGCTCTCGACGAA
*CgPDR1-*R	CTTCCACCATAGTAGCCGCC
*CgERG11-*F	ACCAAGAACAAATGCGCGTC
*CgERG11-*R	GTCCCTTGGGACAACGTAGG
*CgSNQ2-*F	CGATGCACCAACCAAGTATG
*CgSNQ2*-R	ACCACCGACAGTCATCAACA

**Table 2:** Effects of FWE with FLC on eight clinical isolates of *N. glabratus* assessed using the FICI model.

Isolates	MIC Alone (μg/mL)		MIC Combination (μg/mL)		FICI	Interpretation
	FLC	FWE	FLC	FWE		
ATCC 22019	0.5	64	0.25	8	0.625	Addition
Cg1	16	32	8	8	0.75	Addition
Cg2	16	32	8	4	0.625	Addition
Cg3	16	32	4	16	0.75	Addition
Cg4	32	16	16	8	1.0	Indifference
Cg5	16	16	4	8	0.75	Addition
Cg6	16	32	2	16	0.625	Addition
Cg7	8	32	4	8	0.75	Addition
Cg8	8	32	4	8	0.75	Addition

**Table 3:** Effects of FWE with ITR on eight clinical isolates of *N. glabratus* assessed using the FICI model.

Isolates	MIC Alone (μg/mL)		MIC Combination (μg/mL)		FICI	Interpretation
	ITR	FWE	ITR	FWE		
ATCC 22019	0.125	64	0.0625	8	0.625	Addition
Cg1	8	32	1.0 ↓ 8	8	0.375	Synergism
Cg2	8	32	1.0	16	0.625	Addition
Cg3	8	32	1.0 ↓ 8	8	0.375	Synergism
Cg4	4	16	1.0 ↓ 4	8	0.75	Addition
Cg5	1	16	0.25 ↓ 4	8	0.75	Addition
Cg6	0.5	32	0.125 ↓ 4	16	0.75	Addition
Cg7	2	32	0.5 ↓ 4	4 ↓ 8	0.375	Synergism
Cg8	1	32	0.5 ↓ 2	8 ↓ 4	0.75	Addition

The numerical value positioned to the right of the symbol “↓” represents the fold reduction in the MIC relative to monotherapy.

**Table 4:** Effects of FWE with VRC on eight clinical *N. glabratus* isolates assessed using the FICI model.

Isolates	MIC Alone (μg/mL)		MIC Combination (μg/mL)		FICI	Interpretation
	VRC	FWE	VRC	FWE		
ATCC 22019	0.0625	64	0.0313	8	0.625	Addition
Cg1	1	32	0.125 ↓ 8	8 ↓ 4	0.375	Synergism
Cg2	0.5	32	0.125 ↓ 4	8 ↓ 4	0.5	Synergism
Cg3	1	32	0.25	16	0.75	Addition
Cg4	2	16	0.5	8	0.75	Addition
Cg5	0.5	16	0.125 ↓ 4	8	0.75	Addition
Cg6	2	32	0.25 ↓ 8	8 ↓ 4	0.375	Synergism
Cg7	1	32	0.125 ↓ 8	16 ↓ 2	0.625	Addition
Cg8	1	32	0.25 ↓ 4	8 ↓ 4	0.5	Synergism

The numerical value positioned to the right of the symbol “↓” represents the fold reduction in the MIC relative to monotherapy.

**Table 5:** Efflux level of rhodamine 6G in isolate Cg1 under various treatment conditions.

Groups	Efflux Level (%)	
	Absence of Glucose	Addition of Glucose
Control	0.8	9.1
FWE	0.6	16.0
ITR	1.6	17.3
VRC	0.6	17.5
FWE + ITR	0.6	11.4
FWE + VRC	0.6	14.6

## Data Availability

The original contributions presented in this study are included in the article/Supplementary Materials. Further inquiries can be directed to the corresponding authors.

## Supplementary Materials

The following supporting information can be downloaded at: https://www.mdpi.com/article/10.3390/pathogens14040351/s1, Figure S1: Determining rhodamine 6G uptake by cg1 during energy depletion; Figure S2: Determining rhodamine 6G absorption post-cell washing; Figure S3: Assessment of Rhodamine 6G Efflux Following Incubation with Glucose.
