# Supplementary material for: Promising Role of Fruitless Wolfberry Bud Tea in Combating Nakaseomyces glabratus Resistance
Source: Pathogens. 2025 Apr 4;14(4):351. doi: 10.3390/pathogens14040351 (PMC12030577; doi:10.3390/pathogens14040351)
Supplement: Supplementary file 1 [file pathogens-14-00351-s001.zip › pathogens-3500040-supplementary.pdf]

Supporting Information

Promising Role of Fruitless Wolfberry Bud Tea in Combating *Nakaseomyces glabratus* Resistance

Liping Zhang [1], Zhiyan Ma [2], Xuezhang Zhou [2], Ziping Zhang [2,*,†] and Tao Wu [1,*,†]

1 Department of Clinical Laboratory Medicine, People's Hospital of Ningxia Hui Autonomous Region, Ningxia Medical University, Ningxia Hui Autonomous Region, Yinchuan 750002, China; zhanglipingxly@163.com

2 College of Life Science, Key Laboratory of Ministry of Education for Protection and Utilization of Special Biological Resources in Western China, Ningxia University, Yinchuan 750021, China; amzy123456782020@outlook.com (Z.M.); zhouxuezhang@nxu.edu.cn (X.Z.)

* Correspondence: zipingzhang@163.com (Z.Z.); wutao@nxmu.edu.cn (T.W.)

† These authors contributed equally to this work.

Figure S1. Determining rhodamine 6G uptake by cg1 during energy depletion.

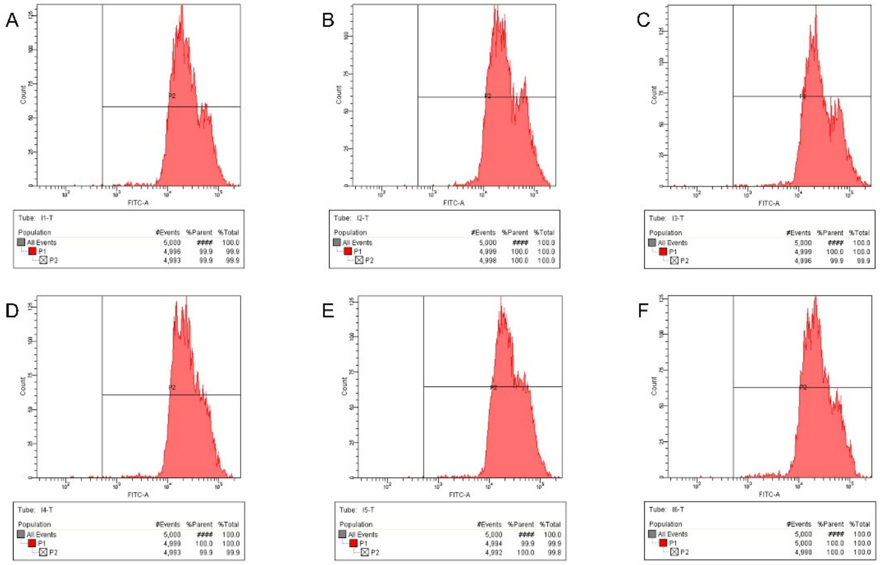

Following a 4-hour depletion of *Candida glabrata*, rhodamine 6G at a concentration of 0.017 mol/L was introduced and co-incubated for an additional 2 hours. The fluorescence intensity of the uptake was subsequently quantified using flow cytometry. (A) control, (B) FWE, (C) ITR, (D) VRC, (E) FWE + ITR, and (F) FWE + VRC.

Figure S2. Determining rhodamine 6G absorption post-cell washing.

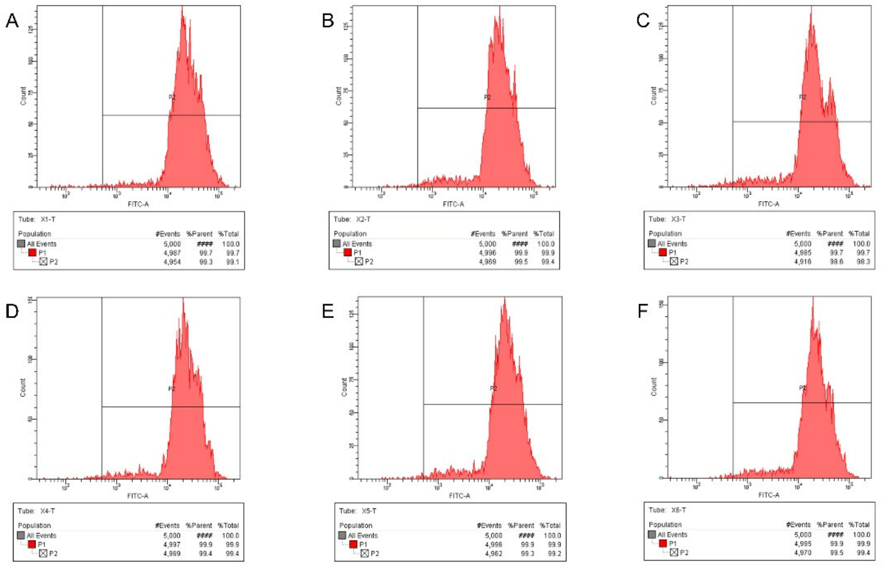

Measuring fluorescence after removing extracellular rhodamine 6G using PBS. (A) control, (B) FWE, (C) ITR, (D) VRC, (E) FWE + ITR, and (F) FWE + VRC.

Figure S3. Assessment of Rhodamine 6G Efflux Following Incubation with Glucose.

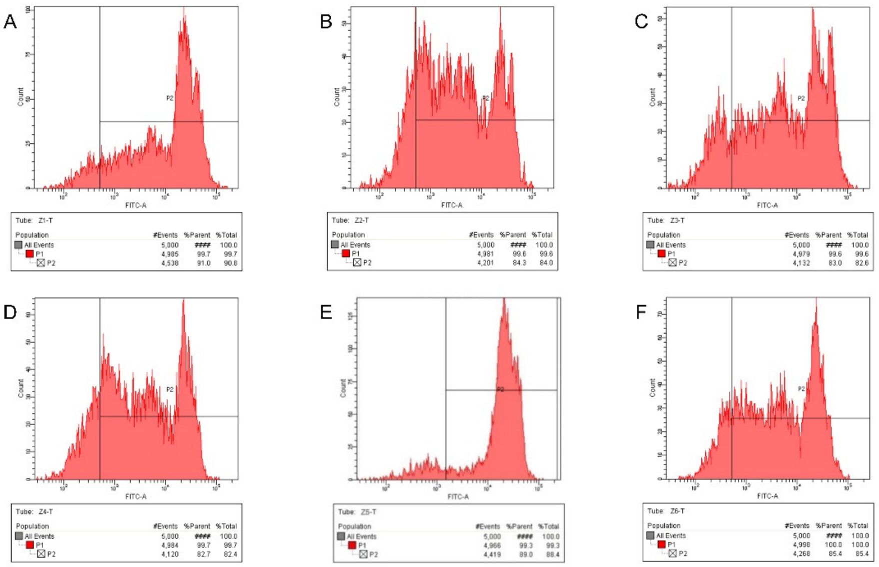

Fluorescence was measured following incubation in phosphate-buffered saline (PBS) containing glucose at 30 °C with agitation at 200 revolutions per minute (rpm) for a duration of 2 hours. (A) control, (B) FWE, (C) ITR, (D) VRC, (E) FWE + ITR, and (F) FWE + VRC.
